# Association between chronic diseases, multimorbidity and insufficient physical activity among older adults in southern Brazil: a cross-sectional study

**DOI:** 10.1590/1516-3180.2020.0282.R1.15092020

**Published:** 2020-12-14

**Authors:** Roselaine da Silva Gomes, Aline Rodrigues Barbosa, Vandrize Meneghini, Susana Cararo Confortin, Eleonora d’Orsi, Cassiano Ricardo Rech

**Affiliations:** I BSc. Master’s Student, Postgraduate Program in Physical Education, Universidade Federal de Santa Catarina (UFSC), Florianópolis (SC), Brazil.; II PhD. Associate Professor, School of Sports, Universidade Federal de Santa Catarina (UFSC), Florianópolis (SC), Brazil.; III MSc. Doctoral Student, Postgraduate Program in Physical Education, Universidade Federal de Santa Catarina (UFSC), Florianópolis (SC), Brazil.; IV PhD. Postdoctoral Researcher, Center for Biological and Health Sciences, Universidade Federal do Maranhão (UFMA), São Luís (MA), Brazil.; V MD, PhD. Associate Professor, School of Health Sciences, Universidade Federal de Santa Catarina (UFSC), Campus Trindade, Florianópolis (SC), Brazil.; VI PhD. Adjunct Professor, School of Sports, Universidade Federal de Santa Catarina (UFSC), Florianópolis (SC), Brazil.

**Keywords:** Motor activity, Aging, Cross-sectional studies, Morbidity, Motor activities, Self-rated health, Elderly

## Abstract

**BACKGROUND:**

Being active has been shown to have beneficial effects for the health of individuals with chronic diseases. However, data on the association between multimorbidity and physical activity are limited.

**OBJECTIVE:**

To investigate the association between chronic diseases, multimorbidity and insufficient physical activity among older adults in southern Brazil, according to sex.

**DESIGN AND SETTING:**

Cross-sectional population-based and household-based study derived from the second wave (2013-2014) of the EpiFloripa Aging Cohort Study.

**METHODS:**

Insufficiency of physical activity (outcome) was ascertained using the long version of the International Physical Activity Questionnaire (≤ 150 minutes/week). Eleven self-reported chronic diseases were identified. Multimorbidity was defined from the number of chronic diseases (none; 2 or 3; or 4 or more). The adjustment variables were age, schooling, marital status, income, smoking, alcohol consumption and cognition. Additionally, each chronic disease was adjusted for the others. Associations were tested using logistic regression (crude and adjusted).

**RESULTS:**

Among the 1197 participants (≥ 63 years), women (54.0%) were more likely than men (39.6%) to be insufficiently active. In the adjusted analysis, women and men with depressive symptoms, and men with diabetes, were more likely to be insufficiently active than those without symptoms. Multimorbid women were more likely to be insufficiently active, and the magnitude of the effect was strongest for 4 or more diseases.

**CONCLUSION:**

This study indicates that the associations were sex-specific. Depressive symptoms and multimorbidity were associated with insufficient physical activity among women, while diabetes was associated with insufficient physical activity among men.

## INTRODUCTION

Population aging is a worldwide phenomenon that has led to increased prevalence of noncommunicable chronic diseases (NCDs). Among chronic diseases, cardiovascular diseases, cancers, respiratory diseases and diabetes are responsible for more than 80% of deaths worldwide.^1^ In 2016, these diseases accounted for 73.8% of deaths in Brazil.^2^ In addition to these diseases, musculoskeletal disorders and neurological and mental disorders are also prevalent.^3^

With the increasing prevalence of NCDs, another challenge for the healthcare of the older adult population is the coexistence of two or more chronic conditions in the same individual, called multimorbidity.^4^ Multimorbidity is an important condition among older adults, and is often associated with disability and a higher chance of mortality. Also, multimorbidity has a higher cost, with greater utilization of healthcare services than would be expected from the individual effects of chronic diseases.^5^ In Brazil, data from the 2013 National Health Survey showed that the prevalence of multimorbidity among men and women aged 60 or over was 43.4% and 57.1%, respectively.^6^

Regular practicing of physical activity stands out as a modifiable risk factor in relation to prevention and control of chronic diseases, and to improvement of the health and wellbeing of individuals or communities.^7^ The World Health Organization (WHO)^8^ recommends that individuals should accumulate at least 150 minutes per week of moderate to vigorous-intensity physical activity or 75 minutes per week of vigorous physical activity. According to data from populational studies focusing on older adults (60 years and over), insufficient levels of physical activity are highly prevalent in Brazil, particularly among women and older age groups.^9^^,^^10^

Many studies have investigated the association between chronic disease and physical activity among older adults. However, these studies were based on a single-disease model,^11^^,^^12^ without taking into account adjustments for other diseases, which is the model proposed in the present study.

Regarding the association between multimorbidity and physical activity among older adults, the data are limited and divergent, including in Brazil, thus suggesting that there is a need for further studies. While some studies have shown an association between multimorbidity and lower levels of physical activity among young adults, middle-aged adults and older adults,^13^^,^^14^ others have found an association only for men (≥ 65 years)^15^^,^^16^ or no association for adults.^17^ Recently, Christofoletti et al.^18^ investigated the association between multimorbidity and physical activity among Brazilian adults (≥ 18 years). However, their study involved different clusters of only four diseases and the analyses were not stratified according to gender and age group.

## OBJECTIVE

Given the above, and considering the differences between men and women and the scarcity of data from the elderly population, the aim of the present study was to investigate the association between chronic diseases, multimorbidity and insufficient physical activity among older adults in southern Brazil, according to sex.

## METHODS

### Data source and study population

This cross-sectional epidemiological study consisted of an analysis on data from the second wave of the EpiFloripa Aging Cohort Study, conducted in 2013-2014. The baseline for this study was in 2009-2010. The EpiFloripa Aging Cohort Study is a prospective population-based and household-based cohort study carried out among older adults (≥ 60 years) living in the urban area of Florianopolis, state of Santa Catarina, in southern Brazil (http://www.epifloripa.ufsc.br/).

Information regarding the data collection, population and sampling process was published previously and is briefly presented here.^19^^,^^20^ In the baseline study (2009-2010), a random sample of 1702 individuals (aged 60 years or over) was interviewed. The second wave of data was collected between November 2013 and November 2014, when the older adults of the baseline study had reached ages of 63 years or over. From this sample, eligibility for inclusion in the second wave took into account deaths and address changes. It was found that 217 deaths had occurred. There were 129 refusals to participate and 159 other losses, among which 111 were due to impossibility of localization. Thus, the sample for the present study was 1,197 interviewees.

This project was approved by the Human Research Ethics Committee of Universidade Federal de Santa Catarina (protocol number 329,650, issued on July 8, 2013; CAAE: 16731313.0.0000.0121). The participants signed an informed consent statement.

### Physical activity

The subjects’ practices of physical activity during leisure time and for transportation were investigated through face-to-face interviews using the long version of the International Physical Activity Questionnaire, as validated for Brazilian elderly people.^21^ The physical activity level was determined based on a score expressed as minutes/week in each domain (leisure-time physical activity and transportation physical activity). These scores were calculated by summing the number of minutes spent in doing moderate activities plus the number of minutes spent in doing vigorous activities (which was multiplied by two), as recommended by the WHO.^8^ These summed times spent on physical activity during leisure and for transport/traveling were categorized as either > 150 minutes/week, which was defined as sufficient level of physical activity or ≤ 150 minutes/week, which was defined as insufficient level of physical activity.^8^

### Chronic diseases

The following chronic diseases were identified (yes or no) through the responses to a simple question (“Has a doctor or a healthcare professional ever said that you have …?): arthritis/rheumatism/arthrosis, cancer, diabetes mellitus, bronchitis or asthma, spinal disease, hypertension, coronary disease, chronic renal failure and/or cerebrovascular disease. These chronic diseases were recorded based on the questionnaire used in the National Household Sample Survey (Pesquisa Nacional por Amostra de Domicílios, PNAD).^22^

To evaluate depressive symptoms, the Geriatric Depression Scale (GDS - 15) was used. This provides validated measurements for identifying depressive symptoms among older adults, and it has been translated and validated for use in Brazil.^23^ Interviewees were classified as presenting symptoms of depression using a cutoff point of ≤ 5. Thus, they were deemed not to present any symptoms of depression if a score of ≥ 6 was reached.

### Multimorbidity

To analyze multimorbidity, the total number of diseases was categorized as follows: no multimorbidity (zero and one morbidity); two or three morbidities; or four or more morbidities.

### Adjustment variables

In accordance with evidence from the literature,^13^^,^^24^ the adjustment variables considered as possible confounding factors were the following: age (in years, as a continuous variable); marital status (married/with a partner; single; divorced/separated; or widowed); schooling level (without schooling; 1 to 4 years; 5 to 8 years; 9 to 11 years; or 12 years or more); monthly family income, categorized into quartiles (1st quartile: US$ 304.34; 2nd quartile: US$ 304.35 to US$ 524.45; 3rd quartile: US$ 524.46 to US$ 1,152.00; or 4th quartile: US$ 1,152.01; at the time of data collection, R$ 2.55 (Brazilian reais) was the equivalent of US$ 1.00 (United States dollars)); smoking (never smoked, current smoker or former smoker); and alcohol consumption (consumed or not consumed).^25^ Cognitive status was measured using the Brazilian version of the Mini-Mental State Examination (MMSE),^26^ and the results were presented as the total score.

### Statistical analyses

The descriptive variables were presented as means, standard deviations, frequencies (absolute and relative) and confidence intervals (95% CI). Sex differences were tested using the chi-square test. Crude and adjusted logistic regression analyses were used to test possible associations between insufficient physical activity and each chronic disease.

In the adjusted analyses, three regression models were considered. In model 1, the analyses were adjusted for sociodemographic characteristics (age, schooling, marital status and income). Model 2 additionally included behavioral characteristics (smoking and alcohol consumption) and cognitive status. Lastly, model 3 additionally included all other chronic diseases, in order to eliminate the potential confounding effect of comorbidity. All variables were maintained in the analyses, regardless of statistical significance.

Logistic regression was also used to test the possible associations between multimorbidity (reference category: 0-1 morbidity) and insufficient physical activity. These analyses were adjusted for models 1 and 2.

All the analyses were stratified according to sex. The analyses were performed using the IBM SPSS Statistics for Windows software (version 22; IBM Corp., Armonk, NY, United States). The logistic regression analyses considered the sample plan (using the “complex sample” module). A significance level of 5% (P < 0.05) was adopted.

## RESULTS

The sample of the present study consisted of 1,197 individuals (778 women). The average age of the women was 74.2 years (± 7.4); and of the men, 73.3 years (± 7.3) (P = 0.037). The mean MMSE score was higher among the men (25.1 ± 5.6) than among the women (24.3 ± 5.7) (P = 0.008). The mean body mass index (BMI) values were 28.5 kg/m^2^ (± 5.4) for the women and 27.0 kg/m^2^ (± 4.1) for the men (P ≤ 0.001).

Table 1 presents the characteristics of the study sample. There were sex differences in all characteristics except for the presence of bronchitis or asthma, and cardiovascular disease. The women were more often insufficiently active (54.0%) than the men (39.6%). The prevalence of married status was higher among the men (83.0%) than among the women (39.8%). The women had lower education and lower household income than the men. The men had higher frequencies of alcohol intake and tobacco use than the women.

**Table 1:** Distribution of older adults in Florianopolis (2013-2014), stratified by sex, according to the characteristics analyzed in the study.

Characteristics		Women (n = 778)			Men (n = 419)			P-value
		n	%	95% CI	n	%	95% CI	
**Physical activity**								
	Sufficient	358	46.0	42.5-49.5	253	60.4	55.6-65.0	**< 0.001**
	Insufficient	420	54.0	50.5-57.5	166	39.6	35.0-44.0	
**Marital status**								
	Married	310	39.8	36.4-43.3	348	83.1	79.1-86.4	**< 0.001**
	Single	62	8.0	6.3-10.1	14	3.3	2.0-5.6	
	Divorced/separated	60	7.7	6.0-9.8	27	6.4	94.4-9.2	
	Widowed	346	44.5	41.0-48.0	30	7.2	5.0-10.0	
**Schooling (years)**								
	< 1	60	7.7	6.0-9.8	33	7.9	5.6-10.9	**< 0.001**
	1 to 4	305	39.4	36.0-42.8	125	29.8	25.6-34.4	
	5 to 8	135	17.4	14.9-20.2	64	15.2	12.1-19.1	
	9 to 11	123	15.9	13.5-18.6	57	13.6	10.6-17.2	
	≥ 12	152	19.6	17.0-22.6	140	33.5	29.0-38.1	
**Income**								
	Quartile 4	171	22.0	19.2-25.1	127	30.4	26.1-35.0	**0.001**
	Quartile 3	206	26.5	23.5-29.7	93	22.2	18.5-26.5	
	Quartile 2	169	21.8	19.0-24.8	104	24.9	20.9-29.3	
	Quartile 1	231	29.7	26.6-33.0	94	22.5	18.7-26.7	
**Living arrangement**								
	With someone	582	74.8	71.6-77.7	374	89.3	85.9-92.0	**< 0.001**
	Alone	196	25.2	22.3-28.4	45	10.7	8.1-14.1	
**Smoking**								
	Never smoked	586	75.4	72.3-78.3	145	34.6	30.2-39.3	**< 0.001**
	Smoked and stopped	149	19.2	16.5-22.1	233	55.6	50.8-60.3	
	Current smoker	42	5.4	4.0-7.2	41	9.8	7.3-13.0	
**Alcohol consumption**								
	No	570	73.3	70.0-76.3	180	42.9	38.2-47.8	**< 0.001**
	Yes	208	26.7	23.7-30.0	239	57.1	52.2-61.7	
**Functional disability**								
	None	196	25.2	22.3-28.4	171	41.1	36.4-45.9	**< 0.001**
	1 - 3	305	39.3	35.9-42.7	149	35.8	31.3-40.6	
	≥ 4	276	35.5	32.2-39.0	96	23.1	19.3-27.4	
**Depressive symptoms**								
	No	571	78.0	74.8-80.9	336	84.4	80.5-97.7	**0.010**
	Yes	161	22.0	19.1-25.1	62	15.6	12.3-19.5	
**Spinal disease**								
	No	348	44.7	41.3-48.2	252	60.1	55.3-64.7	**< 0.001**
	Yes	430	55.3	51.7-58.7	167	39.9	35.2-44.6	
**Arthritis/rheumatism/arthrosis**								
	No	458	58.9	55.4-62.3	328	78.3	74.0-82.0	**< 0.001**
	Yes	320	41.1	37.7-44.6	91	21.7	18.0-25.9	
**Cancer**								
	No	708	91.0	88.8-92.8	351	83.8	79.9-87.0	**< 0.001**
	Yes	70	9.0	7.2-11.2	68	16.2	13.0-20.0	
**Diabetes**								
	No	563	72.4	69.1-75.4	333	79.5	75.3-83.1	**0.007**
	Yes	215	27.6	24.6-30.9	86	20.5	16.9-24.7	
**Bronchitis or asthma**								
	No	633	81.4	78.5-83.9	352	84.1	80.2-87.2	0.253
	Yes	145	18.6	16.0-21.5	67	15.9	12.8-19.8	
**Coronary disease**								
	No	530	68.1	64.8-71.4	276	65.9	61.2-70.3	0.428
	Yes	248	31.9	28.6-35.2	143	34.1	29.7-38.8	
**Chronic renal failure**								
	No	751	96.5	95.0-97.6	391	93.3	90.5-95.3	**0.011**
	Yes	27	3.5	2.4-5.0	28	6.7	4.6-9.5	
**Cerebrovascular disease**								
	No	701	90.1	87.8-92.0	361	86.2	82.5-89.2	**0.040**
	Yes	77	9.9	8.0-12.2	58	13.8	10.8-17.5	
**Osteoporosis**								
	No	520	66.8	63.4-70.1	392	93.6	90.7-95.5	**< 0.001**
	Yes	258	33.2	29.9-36.6	27	6.4	4.4-9.2	
**Hypertension**								
	No	234	30.1	26.9-33.4	182	43.4	38.7-48.2	**< 0.001**
	Yes	544	69.9	66.6-73.0	237	56.6	51.7-61.2	
**Multimorbidity**								
	0-1	134	18.3	15.5-21.1	125	31.4	26.8-36.0	**< 0.001**
	2-3	302	41.3	37.7-44.8	177	44.5	39.6-49.4	
	4+	296	40.4	36.9-44.0	96	24.1	19.9-28.3	

Note: P-value calculated using chi-square test; P ≤ 0.05 is in bold. CI = confidence interval.

The most prevalent chronic diseases among the women were hypertension (69.9%), spinal disease (55.3%) and arthritis/rheumatism/arthrosis (41.1%). The prevalence of multimorbidity (2-3 and 4 or more diseases) was higher among the women than among the men (Table 1).

The associations between diseases and insufficient physical activity are presented in Tables 2 and 3, for women and men, respectively. For the women, the data from the crude analysis (Table 2) show that the diseases positively associated with insufficient physical activity were the presence of depressive symptoms, arthritis/rheumatism/arthrosis, diabetes, cardiovascular disease, cerebrovascular disease and hypertension. After adjusting for models 1 and 2, only the presence of depressive symptoms and cardiovascular disease maintained associations with insufficient physical activity, although the strength of association was reduced. After further adjustment for coexisting diseases (final model), only the presence of depressive symptoms remained positively associated with insufficient physical activity. Thus, the women with depressive symptoms were 2.8 times (95% CI: 1.6-4.7) more likely to have insufficient physical activity than were their peers.

**Table 2:** Crude and adjusted logistic regression analyses on the association between each chronic disease and insufficient physical activity among women.

Diseases	Crude analysis OR (95% CI)	Model 1 OR (95% CI)	Model 2 OR (95% CI)	Model 3 OR (95% CI)
Depressive symptoms	**3.5 (2.1-5.7)**	**3.3 (2.0-5.5)**	**3.0 (1.8-5.0)**	**2.8 (1.6-4.7)**
Spinal disease	1.2 (0.8-1.6)	1.2 (0.8-1.7)	1.1 (0.7-1.7)	1.0 (0.6-1.6)
Arthritis/rheumatisms/arthrosis	**1.5 (1.0-2.1)**	1.3 (0.9-2.0)	1.5 (1.0-2.2)	1.4 (0.9-2.1)
Cancer	1.0 (0.6-2.0)	1.2 (0.6-2.5)	1.4 (0.7-2.8)	1.2 (0.6-2.6)
Diabetes	**1.4 (1.0-2.1)**	1.4 (0.9-2.0)	1.3 (0.9-2.0)	1.0 (0.7-1.7)
Bronchitis	1.4 (0.9-2.3)	1.4 (0.9-2.1)	1.2 (0.8-1.9)	1.1 (0.7-1.7)
Cardiovascular disease	**2.0 (1.4-2.9)**	**1.6 (1.1-2.5)**	1.6 (1.1-2.5)	1.5 (0.9-2.4)
Chronic kidney failure	0.8 (0.4-1.8)	0.8 (0.4-1.6)	0.6 (0.3-1.4)	0.5 (0.2-1.1)
Cerebrovascular disease	**2.1 (1.1-3.8)**	1.5 (0.7-3.1)	1.3 (0.6-2.7)	0.9 (0.4-2.2)
Osteoporosis	1.3 (0.9-1.9)	1.2 (0.8-1.8)	1.2 (0.8-1.8)	1.0 (0.7-1.7)
Hypertension	**1.6 (1.1-2.3)**	1.3 (0.9-1.8)	1.3 (0.9-1.9)	1.1 (0.7-1.6)

OR = odds ratio; CI = confidence interval. Model 1: Adjusted for age, schooling, marital status, living arrangement and income. Model 2: Adjusted for model 1 + smoking, alcohol consumption, cognition, body mass index and functional disability. Model 3: Adjusted for previous models and other chronic diseases.

**Table 3:** Crude and adjusted logistic regression analyses on the association between each chronic disease and insufficient physical activity among men.

Diseases	Crude analysis OR (95% CI)	Model 1 OR (95% CI)	Model 2 OR (95% CI)	Model 3 OR (95% CI)
Depressive symptoms	**3.2 (1.4-7.1)**	**3.0 (1.5-5.8)**	**2.5 (1.2-5.1)**	**2.7 (1.2-5.7)**
Spinal disease	0.9 (0.5-1.6)	0.8 (0.5-1.4)	0.8 (0.5-1.4)	0.8 (0.5-1.3)
Arthritis/rheumatisms/arthrosis	1.5 (0.9-2.7)	1.4 (0.8-2.5)	1.4 (0.8-2.6)	1.5 (0.9-2.8)
Cancer	1.1 (0.5-2.4)	1.0 (0.5-2.2)	1.1 (0.5-2.4)	1.1 (0.5-1.9)
Diabetes	1.5 (0.8-2.7)	1.7 (0.8-3.3)	**2.0 (1.0-3.8)**	**2.2 (1.1-4.3)**
Bronchitis	1.5 (0.7-3.0)	1.4 (0.7-2.8)	1.4 (0.7-2.7)	1.0 (0.5-2.0)
Cardiovascular disease	1.2 (0.7-2.0)	1.2 (0.7-1.9)	1.1 (0.6-1.9)	1.1 (0.6-2.1)
Chronic kidney failure	1.7 (0.6-5.2)	1.6 (0.5-5.0)	1.9 (0.6-6.2)	1.6 (0.4-5.8)
Cerebrovascular disease	1.8 (0.9-3.6)	1.3 (0.6-2.8)	1.0 (0.5-2.3)	0.7 (0.3-1.7)
Osteoporosis	1.3 (0.5-3.2)	1.0 (0.3-2.6)	1.0 (0.3-2.8)	0.9 (0.3-3.0)
Hypertension	1.2 (0.7-1.9)	1.0 (0.6-1.8)	1.2 (0.6-2.3)	1.0 (0.5-2.0)

OR = odds ratio; CI = confidence interval. Model 1: Adjusted for age, schooling, marital status, living arrangement and income. Model 2: Adjusted for model 1 + smoking, alcohol consumption, cognition, body mass index and functional disability. Model 3: Adjusted for previous models and other chronic diseases.

For the men (Table 3), the results from the crude analysis showed that the presence of depressive symptoms was positively associated with insufficient physical activity (odds ratio, OR: 3.2; 95% CI: 1.4-7.1). After adjusting for model 1, the association remained significant. After adjusting for the characteristics in model 2, the association remained significant (OR: 2.5; 95% CI: 1.2-5.1) and diabetes (OR: 2.0; 95% CI: 1.0-3.8) became significant. After further adjustment for coexisting diseases (model 3), depressive symptoms (OR: 2.7; 95% CI: 1.2-5.7) and diabetes (OR: 2.2; 95% CI: 1.1-4.3) retained their positive association with insufficient physical activity.

Table 4 shows the results from the crude and adjusted logistic regression analyses for the association between multimorbidity and insufficient physical activity. For the women, the results from the crude analysis showed positive associations between multimorbidity and insufficient physical activity, both for two to three chronic diseases (OR: 2.1; 95% CI: 1.3-3.4) and for four or more diseases (OR: 3.9; 95% CI: 2.4-6.3). After adjusting for models 1 and 2, the associations were maintained, although to a lower magnitude, for both categories. The results from the adjusted models suggest that multimorbid women were more likely to be physically inactive. This association was significant both for two to three chronic diseases (OR: 1.7; 95% CI: 1.0-2.8) and for four or more diseases (OR: 2.8; 95% CI: 1.6-4.8)

**Table 4:** Simple and multiple logistic regression analyses on the association test between multimorbidity and insufficient physical activity.

Multimorbidity		Crude analysis OR (95% CI)	Model 1 OR (95% CI)	Model 2 OR (95% CI)
**Women**				
	0-1	1	1	1
	2-3	**2.1 (1.3-3.4)**	**1.9 (1.2, 3.0)**	1.7 (0.9-2.8)
	≥ 4	**3.8 (2.3-6.3)**	**3.1 (1.8-5.2)**	**2.8 (1.6-4.8)**
**Men**				
	0-1	1	1	1
	2-3	1.4 (0.8, 2.5)	1.3 (0.7-2.3)	1.3 (0.7-2.3)
	≥ 4	**3.0 (1.4-6.6)**	2.5 (1.1-5.4)	2.3 (1.0-5.3)

OR = odds ratio; CI = confidence interval. Model 1: Adjusted for age, schooling, marital status, living arrangement and income. Model 2: Adjusted for model 1 + smoking, alcohol consumption, cognition, body mass index and functional disability.

For the men, the results from the crude analysis showed a positive association between multimorbidity (four or more diseases) and insufficient physical activity (OR: 3.0; 95% CI: 1.4-6.6). However, after adjusting for the characteristics of models 1 and 2, the associations were not maintained.

## DISCUSSION

This study investigated the association between chronic diseases, multimorbidity and insufficient level of physical activity among older adults in a city in southern Brazil. The results showed that depressive symptoms and multimorbidity (2-3 and ≥ 4 diseases) were positively associated with insufficient physical activity only for women. For men, depressive symptoms and diabetes were positively associated with insufficient physical activity.

According to the results, there was higher prevalence of insufficient levels of physical activity among women, which is consistent with the literature.^27^^,^^28^ The factors contributing to this profile included the facts that this cohort of older women had had lower levels of schooling, were more involved in household/caring activities and did not have any paid employment.^29^ Thus, the women’s lower income levels, compared with men, along with their sociocultural role and family responsibilities, can hinder their possibilities for engaging in physical activity, in terms of both the time and the expense involved in accessing these activities.^30^ Other factors that can contribute towards women’s lower involvement in physical activity include higher prevalence of physical limitations, lack of confidence and lower levels of self-efficacy beliefs.^31^ In the present study, the women had lower schooling and income levels, had higher prevalence of functional limitations and more often lived alone, without company. These results help to explain the higher prevalence of insufficient physical activity among women.

The association between depressive symptoms and insufficient levels of physical activity that was found in the present study is consistent with previous studies showing an inverse relationship between depressive symptoms and physical activity.^11^^,^^32^ It is known that the association between depressive symptoms and physical activity may be bidirectional. Hence, physical activity is considered to be a healthcare strategy for reducing the risk of developing depression. However, depression is a risk factor for reduction of physical activity levels and sedentary behavior.^11^ It is believed that the characteristics of depressive symptoms, such as apathy, dysphoria, cognitive impairment and feelings of discomfort make it difficult to practice physical activity,^33^ regardless of other comorbidities. Therefore, these are considered to be barriers against practicing physical activity, among individuals with depressive symptoms.^34^

Our results partially agree with those from other studies.^11^^,^^32^ Achttien et al.^11^ showed that there was an association between symptoms of depression and lower levels of physical activity, without any difference between men and women (n = 1250; mean age 73.9 ± 6.9 years). In a study without stratification according to sex, Ludwig et al.^32^ found a positive association between depressive symptoms and insufficient physical activity, regardless of sociodemographic and behavioral factors, or BMI. Depression is more prevalent among individuals with chronic diseases.^35^ In the present study, most of the diseases were more prevalent among women, as was insufficient physical activity.

The association between diabetes and insufficient physical activity found in the present study is consistent with the findings of Hult et al.,^12^ who showed that individuals with known diabetes were less active than were those without diabetes. It is known that regular physical activity is important for blood glucose management and overall health among individuals with diabetes.^7^ However, diabetes-related complications such as peripheral neuropathy, muscle and joint pain, poor eyesight and fatigue can limit involvement in regular physical activity.^36^

The results showed that multimorbidity (2-3 and ≥ 4 diseases) was positively associated with insufficient levels of physical activity only among women, thus suggesting that there was a gender difference in this result. Few studies have explored associations between multimorbidity and physical activity among older adults and the results have been incongruent.^13^^–^^16^ The data from the present study are partly in line with those from other studies that showed an association between multimorbidity (≥ 2 diseases) and lower levels of physical activity.^13^^,^^14^ In a study on 228,024 adults (aged 18 years or over), without stratification according to sex, the results showed that those with multimorbidity were significantly less physically active.^14^ Keats et al.^13^ found associations both for men and for women in a study involving 18,709 participants (aged 35 to 79 years). Unlike in the present study, other authors^15^^,^^16^ identified an association between lower levels of physical activity and multimorbidity among men aged 65 or over.

Some factors make it difficult to compare these studies. The observational nature, outcome and inclusion of older adults in the samples were similar characteristics between the present study and others.^13^^–^^16^ The characteristics of the sample (stratification according to sex and age groups), the number and type of chronic diseases included for the categorization of multimorbidity, the instruments used to verify physical activity and the characteristics used in the fit analysis differed between the studies.

The categorization of multimorbidity may have an impact on the results, given that its prevalence estimate is affected by the number of chronic conditions included in the study, as well as the minimum number of diseases used in the categorization.^37^ In studies evaluating the association between multimorbidity and physical activity, the lists of chronic conditions has ranged from nine^14^ to twenty-three,^17^ while in the present study eleven diseases were considered.

Most studies have categorized multimorbidity as the presence of two or more diseases.^13^^,^^14^^,^^17^ Like in the present study, Hudon et al.^17^ defined multimorbidity as the presence of two or more diseases in individuals; however, they also used other cutoff points for analyses (two, three, four and five or more diseases). Cimarras-Otal et al.^16^ took the definition of multimorbidity to be the presence of three or more diseases in the individual, with the justification that the threshold of three or more diseases might provide greater specificity than just two or more. In the present study, four or more diseases was found to have the highest prevalence. This was also the category with the greatest strength of association with insufficient physical activity among women, which highlights the importance of considering other cutoff points for studies on multimorbidity. It is also important to note that the relationship between physical activity and multimorbidity can be bidirectional.^28^ Thus, people with multimorbidity may be less physically active due to worse health conditions. On the other hand, worse health conditions resulting from multimorbidity may lead to insufficient physical activity.

The present study had several limitations. These included the self-reporting of chronic diseases, which may have given rise to memory bias, and the fact that the severity and type of disease were not taken into consideration. Because our definition of multimorbidity was limited to self-reported diseases, these counted equally and their severity was not identified. Moreover, although we made adjustments for some important potential confounders, there may still have been some residual confounding. On the other hand, even though our use of the instrument for investigating physical activity (IPAQ) can be seen as a limitation due to the self-reported nature of the data, this instrument has been validated and is widely used in epidemiological studies.^13^^,^^14^^,^^16^

The strengths of this study include its representative sample and the methodological rigor of the EpiFloripa Aging Cohort Study, including the procedures used in the data collection, training of interviewers, standardized measurements and validated questionnaires. Stratification according to sex is another strong point of the present study, given that there are differences between men and women, especially regarding health conditions and physical activity.^9^^,^^38^ Moreover, our study analyzed older adults’ data and, prior to this, only limited data on the association between chronic diseases, multimorbidity and insufficient levels of physical activity among older adults had been available.

## CONCLUSION

This study showed that the prevalence of insufficient levels of physical activity differs between the sexes, such that this prevalence is higher among females. It also indicated that sex-specific associations exist. Depressive symptoms and multimorbidity were associated with insufficient physical activity among women, while diabetes was associated with insufficient physical activity among men.

From the results obtained and the known benefits of physical activity, the need for longitudinal studies and interventions to better investigate the relationship between chronic diseases, multimorbidity and insufficient physical activity can be emphasized. These should include investigation of the severity of diseases and use of direct measurements of physical activity. Promotion of policies to encourage regular physical activity for individuals with chronic diseases, along with intervention programs, should be differentiated for men and women, and should take into account disease characteristics and individuals’ limitations.
